# Damage by typhoon Hato compared among three different plant communities in Macau, China

**DOI:** 10.1002/ece3.10574

**Published:** 2023-10-05

**Authors:** Qifei Yi, Wen Ye, Faguo Wang, Fuwu Xing, AJ Harris, Lei Duan, Hongfeng Chen

**Affiliations:** ^1^ Key Laboratory of Plant Resources Conservation and Sustainable Utilization, South China Botanical Garden Chinese Academy of Sciences Guangzhou China; ^2^ Key Laboratory of Ministry of Education for Coastal and Wetland Ecosystems, School of Life Sciences Xiamen University Xiamen China

**Keywords:** community structure, diameter at breast height, succession, tree layers, tropical cyclone, wind damage

## Abstract

Tropical cyclones are among the major climatic disasters threatening human survival and development. They are also responsible in part for forest taxonomic composition and dynamics and may lead to catastrophic succession between ecosystems. In this study, we aimed to investigate the extensiveness and severity of the effect caused by Typhoon Hato among the three primary plant communities in Macau, China, including Guia Hill, Taipa Grande, and Ka Ho. The plants' damage was classified into seven categories, ranging from Degree 6, which represents the most severe damage, to Degree 0, which represents almost no damage. The impact of Typhoon Hato was evaluated at different levels, including sample plots, species, DBH, and community structure. Our results show that the sub‐climax community of Guia Hill was most disturbed, with the highest damage index (DI) of 55.28%. Similarly, the Ka Ho shoreline shrub community was also considerably influenced, with a DI of 48.14%. By contrast, the managed secondary forest around Taipa Grande was the least affected, with a DI of 32.66%. Additionally, from the tree layer perspective, the tall trees at Guia Hill canopy layer were directly affected by wind, while the dense understory layer suffered from severe secondary damage due to the fallen trees and branches. For Taipa Grande, the dominant species in the canopy layer were shorter and had less direct damage; the secondary damage was also small as a consequence. Ka Ho had more dwarfed and multibranched species surviving from the sea breeze since Ka Ho was close to the sea. The dense plant structure in Ka Ho protected plants from being easily broken by typhoons, but some twigs and leaves were lost. Some less damaged local species and easily recovered species found in this study could inform the selection of wind‐resistant species for the typhoon‐affected communities.

## Graphical abstract

The extensiveness and severity of the effect caused by Typhoon Hato among the three primary plant communities in Macau, China, including Guia Hill, Taipa Grande, and Ka Ho was researched in this study. The sub‐climax community of Guia Hill was most disturbed, the Ka Ho shoreline shrub community was also considerably influenced. By contrast, the managed secondary forest around Taipa Grande was the least affected. The representative pictures of overall views and close‐ups of three plots before and after super Typhoon Hato.

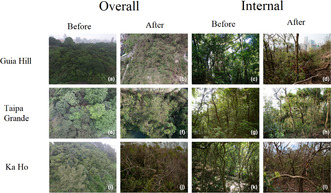

## 1 INTRODUCTION

The intensity and frequency of various natural disasters have increased with changes in the global environment during the Anthropocene (Lewis & Maslin, 2015) and are threatening human survival and development (Rubin, 2014). One of the main natural disasters is wind damage, which results from cyclones, tornadoes and other storm systems (Chen et al., 2013; Foster & Boose, 1992; Gresham et al., 1991; Szwagrzyk et al., 2017; Yasuhara et al., 2012). In storm‐prone areas, wind damage is also one of the major factors affecting forest composition and dynamics (Attiwill, 1994; Foster, 1988; Shifley et al., 2000; Zong et al., 2018). Thus, considerable research has been focused on catastrophic wind events that can result in the successional change of an ecosystem (Chirici et al., 2018; Yamashita et al., 2002).

Typhoons are one of the main climatic disasters in coastal areas of tropical and subtropical zones (Tong & Yang, 2007). These tropical cyclones occurring in the Northwest Pacific Ocean combine extremely strong winds with unpredictable heavy rain, causing large‐scale blowdowns in the forests that they strike. Chen et al. (2011) (Niu et al., 2011; Zhang et al., 2017) assessed the hazards, vulnerability, and risk of the China coast to typhoons. The factors causing typhoon disasters are complex and varied, with different trajectories having different influences that lead to significantly different hazards (Huang, 2012; Ye et al., 2014, 2020). Ye et al. (2019) analyzed the exposure of China's coastal area to typhoon disasters based on geospatial parameters and found that the closer to the coastline, the greater the hazards.

Typhoon influences on the structure and dynamics of tropical and subtropical coastal forest ecosystems have long been studied (Bellingham et al., 1995, 1996; Lin, Hamburg, Hsia, & Lin, 2003b; Lin, Hamburg, Tang, et al., 2003a; Liu & Xu, 2020; Sato, 2004; Tong & Yang, 2007; Wang et al., 2003, 2007; Yamashita et al., 2002; Zhou, 1998; Zhou et al., 1996; Zimmerman et al., 1994; Zimmerman & Covich, 2007), with attention also on urban vegetation (Boose et al., 1994; Lin et al., 2006), wind damage to specific tree species (Karl, 2000; Wang et al., 2000), and defensive countermeasures (Chen et al., 1999, 2000; Hou et al., 2011; Huang, 2002; Lee et al., 2005; Yang et al., 2015; Zhang, 2000). For example, Foster (1988) found that at the species level, trees forming the canopy were more vulnerable to severe wind damage than those occurring in the sub‐canopy. Moreover, Tong and Yang (2007) reviewed typhoon damage types and influencing factors for trees and forest stands. They proposed that damage types mainly include uprooting, crown losses, snapped stems, and defoliation, while species canopy position influences the damage degree.

In eastern Asia, about 30% of all typhoon tracks lead to landfall events in China (Zhao et al., 2018) and cause millions to billions of dollars in losses in damage to infrastructure, as well as major disturbances to ecological systems (Li et al., 2021; Lu et al., 2020). Within China, areas along the Pearl River, including Macau, have suffered the most vegetation disturbance since 2000 (Li & Xue, 2018; Lu et al., 2020). On average, there is one typhoon landing in the Pearl River each year and four to five in some years. Thus, the forest communities within Macau are among the most highly affected by typhoon winds within the country. In 2017, Typhoon “Hato” made landfall with measured maximum wind speeds near its eye of up to 45 m/s. It battered Macau with gusts up to 60.4 m/s (217.4 km/h) (http://www.gep.gov.mo/event/). Hato was a severe typhoon (maximum average wind speed of 41.9–50.9 m/s, National Standard for Tropical Cyclone Classification‐GBT‐19201‐2006) and was the most damaging typhoon in terms of strength and economic losses since 1949 (Liu, Chen, et al., 2018; Liu, Fu, et al., 2018). Typhoon Hato caused Macau an estimated economic loss of around MOP 11.47 billion (US$ 1.4 billion), according to the Statistics and Census Service (DSEC) of Macau (https://www.gov.mo/en/news/92355/) (Government of Macau Special Administrative Region Statistics and Census Service, 2012). Many studies have been conducted to determine the cause, path, and characteristics of Typhoon Hato (Zhang, Yi, et al., 2018; Zhang, Shi, & Li, 2018; Qin et al., 2019; Zhang et al., 2019; Huang et al., 2017; Liu, Chen, et al., 2018; Liu, Fu, et al., 2018; Lin et al., 2020). However, the research on typhoon damage to plants and its impact on community structure needs to be strengthened.

Through this study, we explored the differences in storm impacts in three sample plots with different vegetation types. Our main questions were: Do different plant communities and plant species have different degrees of effects from Hato? Are there any plant species that are relatively resistant to wind? The influence of terrain on forest effects was also discussed. This study contributes to the assessment of Typhoon Hato's impacts by analyzing its effects on the forest communities of Macau. We used a forest plot approach to study the three main vegetation communities in Macau: sub‐climax community, shoreline shrub community, and secondary forest community, or Guia Hill, Ka Ho, and Taipa Grande, respectively. We rated damage to individual trees to compare among communities and tree species. Our study provides insights for improving countermeasures for the vegetation of Macau to resist wind damage from future typhoons, which are expected to increase in intensity due to global warming (China Meteorological Administration, 2021). Additionally, our study may be foundational for long‐term phenological monitoring since this study is part of the on‐going phenological monitoring project started from 2011.

## 2 MATERIALS AND METHODS

### 2.1 Study area

Macau consists of the Macau Peninsula and the islands of Taipa and Coloane, which are now connected together by Cotai, an area of land reclamation. Macau covers an area of 32.9 km^2^ in 2020 (data obtained from the map on the governmental website of Macau and the Cadastre) (Cadastre Bureau: http://www.dscc.gov.mo/zh‐hans/geo_statistic_web1.html) with a coastline of 76.70 km. It is located on the west coast of the Pearl River Estuary, between 113°31′33″–113°35′43″ E longitude and 22°06′39″–22°13′06″ N latitude. Macau has a subtropical oceanic climate with 2000 mm of annual average precipitation and a 22.6°C annual average temperature from 1981 to 2010. The rainfall is concentrated in spring and summer, with an average annual relative humidity of 78.8%. The typhoon season in Macau is from May to October, with July to September being the peak months. The terrain of Macau is high in the south and low in the north. The highest point is Coloane Alto on Coloane, with an elevation of 170.60 m. Two other main hills are Colina da Guia (90.00 m) on the Macau Peninsula and Taipa Grande (158.20 m) in Taipa (Xing et al., 2004).

**FIGURE 1:**
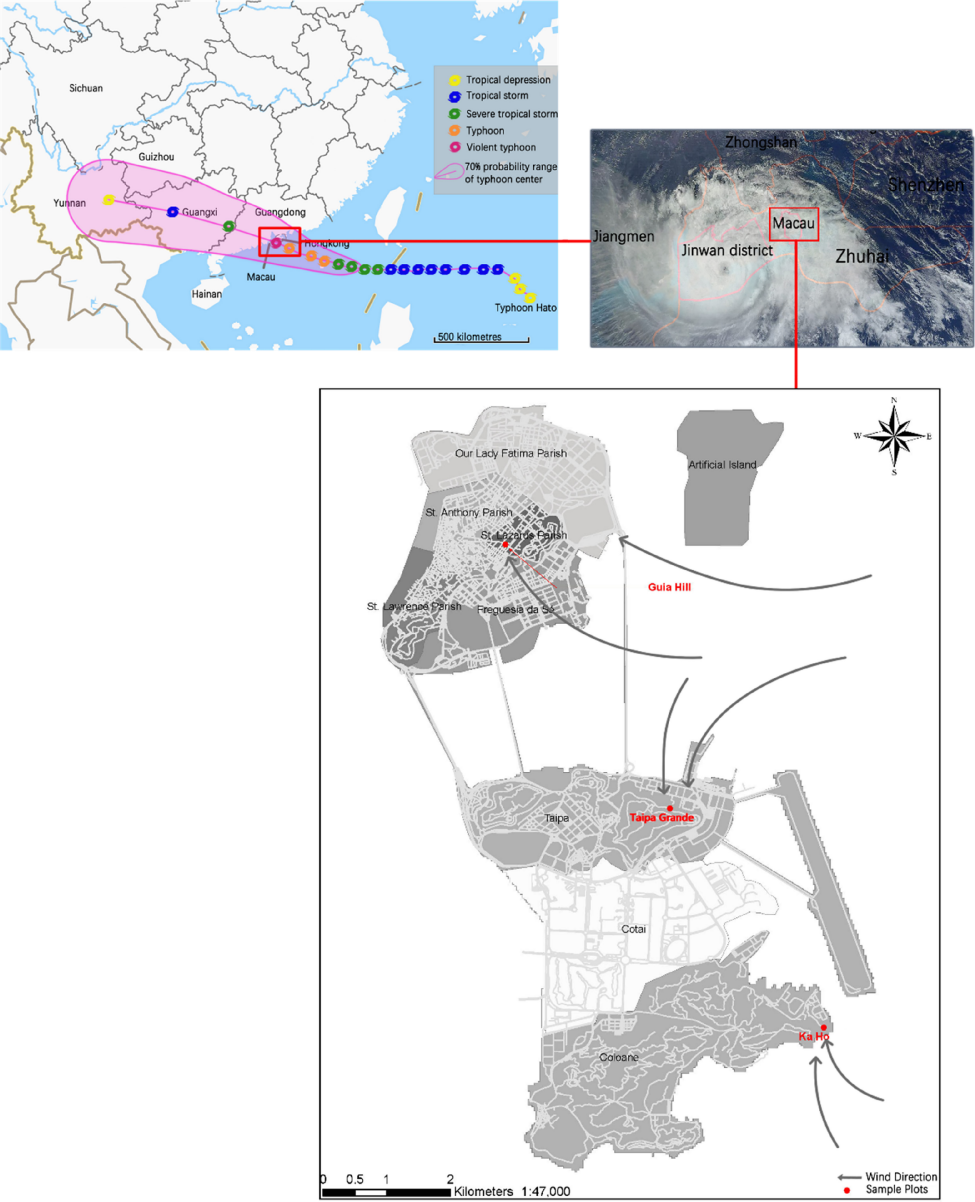
Map of the study area in Macau showing the positions of the plots investigated (Guia Hill, Ka Ho, and Taipa Grande) and the track of Typhoon Hato with relation to the three plots.

In Macau, the subtropical flora shows a close affinity to that of Guangdong. The Flora of Macau (2005–2007) (Xing, 2005) indicates that there are 1508 species of vascular plants belonging to 866 genera and 206 families, among which 812 were native species, belonging to 525 genera of 158 families. Genera and species mentioned throughout the paper use the Flora of China. In this study, we compared three representative communities (Figure 1) selected from among permanent sample plots in Macau (Zhang, Shi, & Li, 2018; Zhang, Yi, et al., 2018). Because these plots were previously established and assessed, it was possible for us to investigate how community characteristics changed after the typhoon. Our investigation of Hato's impacts was carried out on September 11–14, 2017, roughly 1 month following the occurrence of the typhoon. We then conducted another investigation in October 2018, a year after Hato, to observe the community's recovery from the typhoon.

To investigate the effects on forests caused by this extremely strong typhoon, three representative vegetation types in Macau were selected for study. These comprised the sub‐climax community of Guia Hill, representing, in total, an 800 m^2^ (20 m × 40 m) sample area (SA) of the Macau Peninsula, the shoreline shrub community at Ka Ho (400 m^2^ SA, 20 m × 20 m) on Coloane, and the managed secondary forest of Taipa Grande (400 m^2^ SA, 20 m × 20 m) on Taipa. For each community, the sample area was divided into 5 m × 5 m sample plots. There were 32 plots on Guia Hill and 16 plots in Ka Ho and Taipa Grande.

Guia Hill, situated in the middle part of mainland Macau, is the highest point of the peninsula. The vegetation of Guia Hill comprises a more than 100‐year‐old sub‐climax natural community (Table A1). The Guia Hill sample plot (Guia Hill in short) is located at 22°11′51.5″ N latitude, 113°32′47″ E longitude, and 90 m above sea level. The tree density of stems with Diameter at Breast Height (DBH) ≥1 cm, is 7900 trees per km^2^. The slope of the sample area is south by southeast with a gradient of 50°. The upper canopy layer mainly consists of tall tree species like *Cinnamomum burmannii*, *Triadica cochinchinensis*, *Litsea monopetala,* and *Ficus microcarpa*. The sub‐canopy layer mainly includes medium‐ to small‐sized trees, such as *Sterculia lanceolata*, *Mallotus paniculatus*, *Syzygium jambos*, and *Microcos paniculata*. The understory is composed of dwarf shrubs and a few herbs. The main species are *Psychotria asiatica*, *Desmos chinensis*, *F. hirta*, *Pteris semipinnata*, and *Alocasia odora*. Several species of lianas are also present in the community of Guia Hill, such as *Elaeagnus loureiroi*, *Parthenocissus dalzielii*, and *Vitis balansana*.

Taipa Grande is near the highest point of the Taipa Peninsula, and the sample plot (Taipa Grande in short) is located at 22°09′37.15″ N latitude, 113°34′10.67″ E longitude, at 129 m above sea level. At Taipa Grande, the 50–60‐year‐old secondary forest was crafted by humans and is managed for ecological restoration of landfill sites (Table A1). The tree density is 3800 trees per km^2^, lower than that of Guia Hill. The slope is to the southeast with a 40° gradient. At Taipa Grande, the canopy is dominated by *T. sebiferum*, *T. cochinchinensis*, *Schima superba*, *Homalium cochinchinense*, and *S. lanceolata*. The sub‐canopy mainly consists of *S. lanceolata*, *Schefflera heptaphylla*, *Ilex asprella*, *Archidendron lucidum*, *L. rotundifolia* var. *oblongifolia*, and *M. paniculatus*. The lower layer mainly consists of *P. asiatica*, *S. lanceolata*, *Adiantum flabellulatum*, and *Dicranopteris pedata*. There are lianas such as *Callerya nitida*, *Hedyotis hedyotidea*, *Gymnema sylvestre*, *Diploclisia glaucescens*, and *Mussaenda pubescens*.

The Ka Ho sample plot (Ka Ho in short) is located along the eastern shore of Coloane, at 22°07′45.90″ N latitude and 113°35′29.03″ E longitude, with elevation from sea level to 30 m. The vegetation is composed of ca. 50‐year‐old typical shoreline shrub community (Table A1). The tree density at Ka Ho is 9000 trees per km^2^, the slope is to the south–south east, and the gradient is 55°. Trees within this plot are prostrate and intertwine with each other. The main tree species constituting the canopy are *Acacia confusa* and *S. kwangtungense*. The main sub‐canopy trees are *L. rotundifolia* var. *oblongifolia*, *S. heptaphylla*, *Eurya nitida*, and *M. paniculatus*. The understory plants mainly include *P. asiatica* and *Liriope spicata*. The main liana species are *Embelia laeta*, *Gymnema sylvestre*, *Zanthoxylum nitidum*, *M. pubescens*, and *Hypserpa nitida*. *L. rotundifolia* var. *oblongifolia*, and *A. confusa*, which occur throughout the whole community.

### 2.2 Typhoon Hato

Severe Typhoon Hato is the strongest typhoon to affect Macau since 1953. The typhoon (Figure 1) made its landfall in Guangdong province on 23 August 2017, and its closest position to Macau was ca. 40 km south by southwest of the shoreline. In addition to high wind speed, Hato deposited more than 30 mm of average precipitation throughout Macau (Table A2). Moreover, the typhoon happened to coincide with a high tide, thus causing severe damage to plants throughout the area. Numerous trees were blown down or snapped, with broken branches all over the woodland. The forest ecosystem suffered sudden and severe effects.

### 2.3 Data collection

We measured DBH at 1.3 m from the bases of trees, and the trees with DBH ≥ 1 cm were tagged (Chen et al., 2014). For lianas, we measured DBH from the base, and individuals with DBH ≥ 1 cm were also tagged. All tagged plants have been monitored since 2011. We recorded the tagged plants' species, DBH, crown width, tree height, and damage characteristics and took pictures of them, while the damage characteristics were then classified into seven degrees according to the severity of damage as follows:

Degree 6: The base of the main stem broken with no leaves retained, or the whole plant blown down with the root system completely plucked out of the ground;

Degree 5: Main stem broken but with some branches and leaves remaining, or main branches broken with less than 30% of leaves remaining;

Degree 4: Trunk leaning at more than 45° to the ground, or more than 50% of the twigs in the top of the tree crown are broken, but the crown is basically preserved with over 30% of branches and leaves retained;

Degree 3: Trunk leaning at angles between 30° and 45°; 30%–50% of the twigs in the tree crown broken while the tree crown retained; over 50% of branches and leaves remained;

Degree 2: Less than 30% of the twigs in the tree crown were broken, the crown was well‐preserved, over 70% of the branches and leaves were remaining, or most branches were unaffected but more than 50% of the leaves fell due to the wind;

Degree 1: Less than 50% of the leaves have fallen or edge damaged by the wind;

Degree 0: Undamaged or almost undamaged.

### 2.4 Data analysis

#### 2.4.1 Sample plots and species damage index

We calculated the average damage degree and index for communities or species using the following formulae to indicate typhoon effects (Liu & Xu, 2020):

$$DR_{n}=\frac{D_{n}}{N}\times 100\%$$

$$\mathrm{DG}=\frac{\sum \left(D_{n}\times G\right)}{N}$$

$$\mathrm{BR}=\frac{D_{4}+D_{5}+D_{6}}{N}\times 100\%$$

$$\mathrm{DI}=\frac{\sum \left(D_{n}\times G\right)}{N\times \mathrm{SC}}\times 100\%$$

DR_n_: Damage rate of Degree *n*; *D*_*n*_: the number of plants belonging to Degree *n*; *N*: The total number of plants in the corresponding community or species; DG: The average degree of wind damage; *G*: Grade of wind damage (Degree 0–6); BR: rate of severe damage caused by Hato; degree of damages above Degree 4; DI: Wind damage index; SC: The highest damage grade (Degree 6).

DR_*n*_ mainly represents the extensiveness of damage, while DG, BR, and DI reflect the severity of damage. These indexes were calculated and compared at the community level and species level (selected species with more than 5 individuals in each plot) to analyze the difference.

The data of all tree damage degrees in Guia Hill, Taipa Grande, and Ka Ho were analyzed with chi‐square Tests and pairwise *Z*‐Tests through SPSS 22 to indicate the difference in Hato influence between sample plots. The number of plants of each damage degree in each sample plot was weighted before the analysis for an accurate result.

### 2.5 Wind damage in each DBH class

To further analyze the damage for plants in different size classes, trees and lianas were divided into eight classes based on DBH and then summarized in three tree layers as follows:

**Table:** 

Tree layer	DBH class
Understory layer	1.0 cm ≤ DBH < 2.5 cm
Sub‐canopy layer	2.5 cm ≤ DBH < 5 cm
	5 cm ≤ DBH < 7.5 cm
Canopy layer	7.5 cm ≤ DBH < 10 cm
	10 cm ≤ DBH < 15 cm
	15 cm ≤ DBH < 20 cm
	20 cm ≤ DBH < 30 cm
	DBH ≥ 30 cm

Furthermore, to find out the details of the severity of damage in each DBH class, we calculated the DBH damage rate (DBH_*n*__DR) as followed:

$$\mathrm{DB}H_{n}\_\mathrm{DR}=\frac{\mathrm{DB}H_{n}\_D_{6}}{\mathrm{DB}H_{n}\_N}$$

DBH_*n*__DR: Damage rate of DBH class *n*, DBH_*n*__D_6_: Number of degree six plants in DBH class *n*, DBH_*n*__*N*: Number of plants in DBH class *n*.

### 2.6 Changes in community composition

The relative importance value (IV) is a comprehensive quantitative index indicating the status and function of species in the community, using the following formula (Fang et al., 2009).

$$\mathrm{IV}=\frac{\left(\mathrm{RBA}+\mathrm{RA}+\mathrm{RF}\right)}{3}$$

RBA: relative breast height sectional area; RA: relative abundance; RF: relative frequency.

We chose the five most important species at each tree layer in each sample plot to compare their IVs before and after the typhoon to get some insights into the change in community composition.

## 3 RESULTS

### 3.1 Comparison of wind effect among three sample plots

**FIGURE 2:**
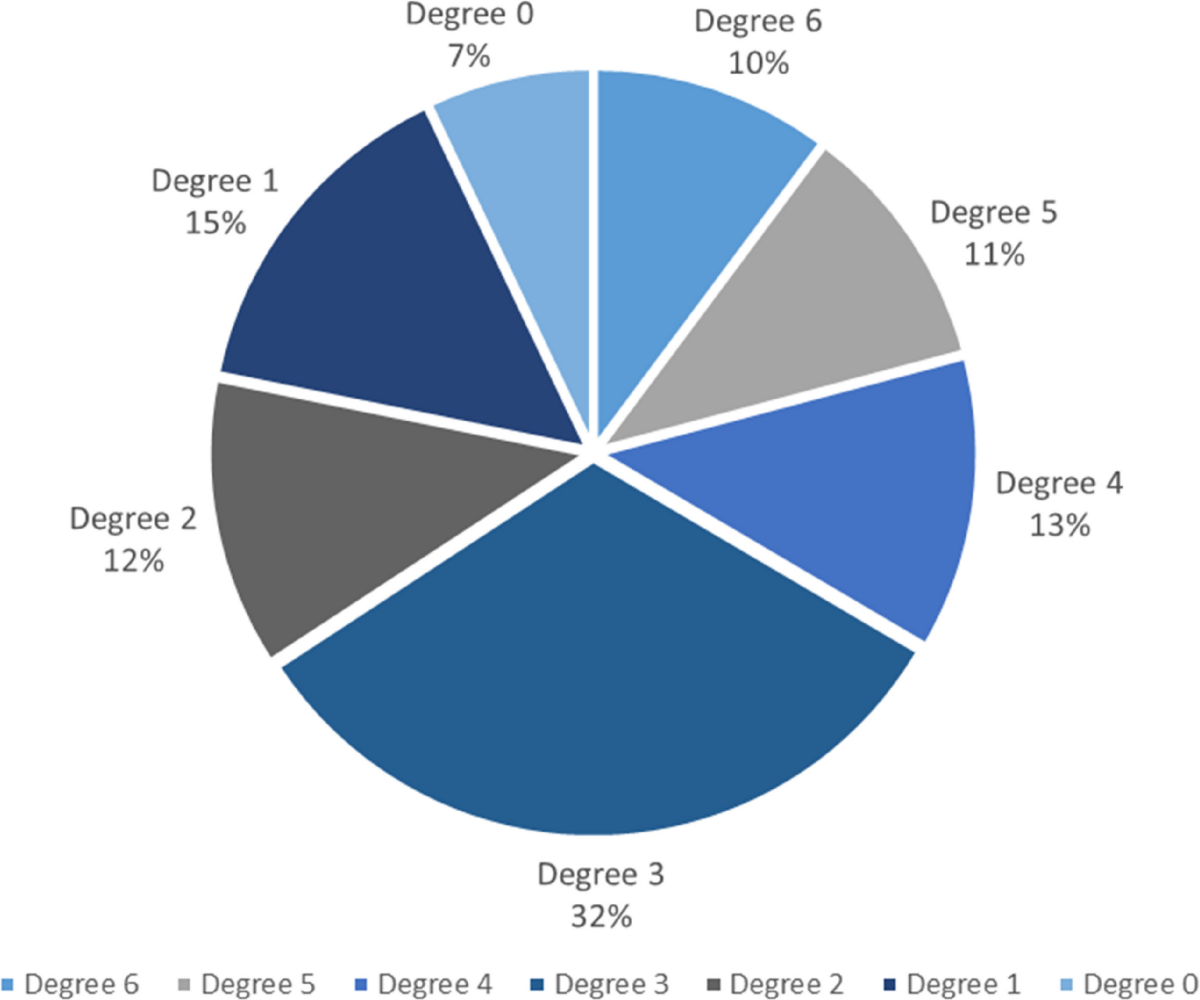
Proportion of wind damage degrees over the 1049 individuals measured in the three forest plots.

**FIGURE 3:**
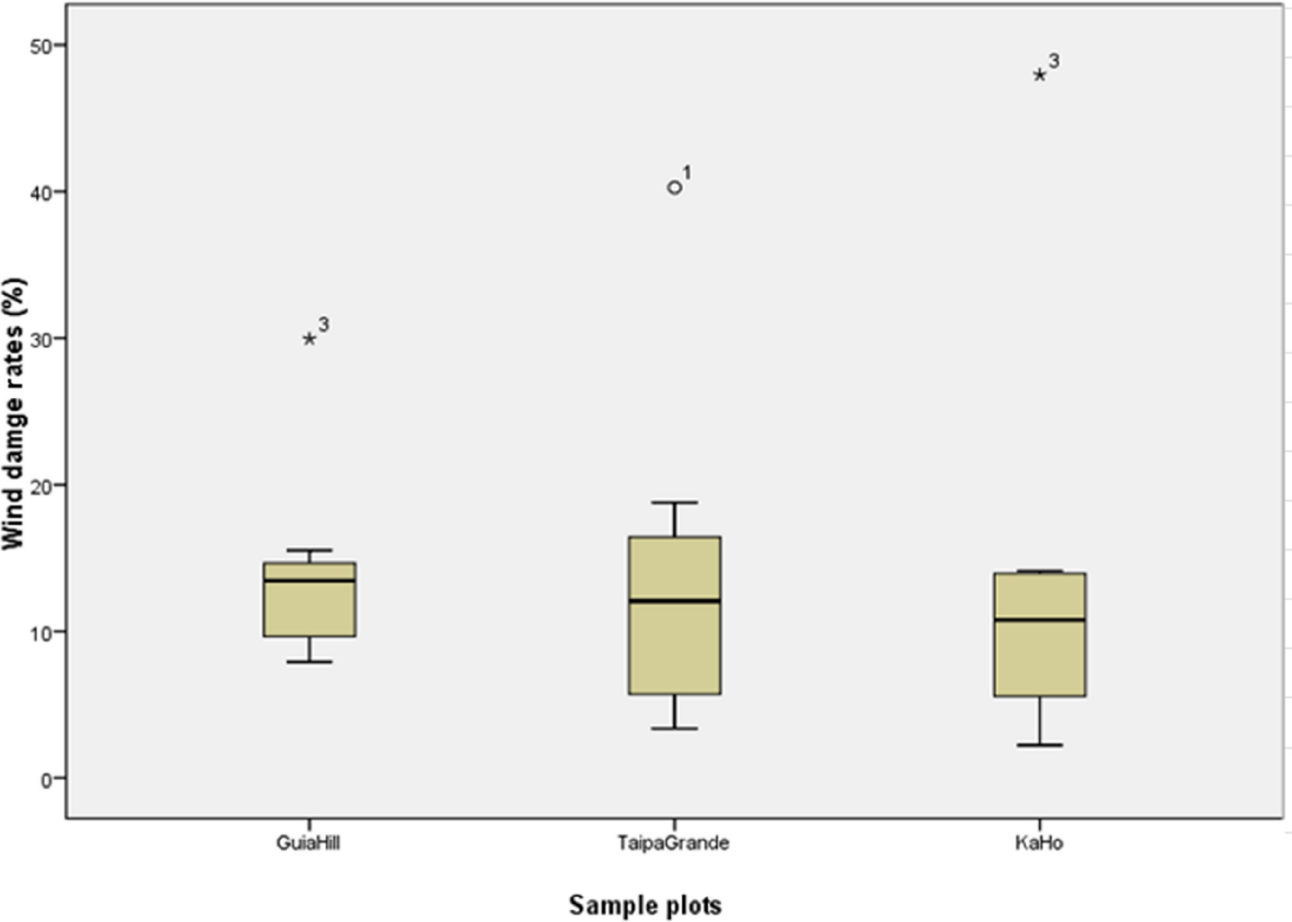
Boxplot of Damage Rate (DR_*n*_) of each degree in the three plots (Guia Hill, Ka Ho, and Taipa Grande). *, o Indicate the outliers of each sample plot. * Indicated DR_3_ was significantly high in Guia Hill and Ka Ho, and o indicated DR_1_ was significantly high in Taipa Grande compared to other DR_*n*_.

In total, we tallied 1049 individual trees belonging to 63 species, 54 genera, and 32 families in three plots before Typhoon Hato. Among the investigated trees in all three plots, Degree 3 accounts for the largest proportion with a DR_3_ of 32% (Figure 2). DR_3_ is significantly higher (shown as outliers in Figure 3) in Guia Hill and Ka Ho, and DR_1_ represents a significant proportion compared to other degrees in Taipa Grande (shown as outliers in Figure 3). Effects varied across the three sample plots (χ^2^ = 185.697, *p* ≤ .01) (Table A3), indicating the influence caused by Hato is significantly different for Guia Hill, Taipa Grande, and Ka Ho. Furthermore, the pairwise *Z*‐Tests (Table A4) showed that the sample plots differed from each other in each specific damage degree. Guia Hill had higher proportions of stems with severe damage (degrees 5 and 6) than the other sites, while Ka Ho had fewer undamaged stems (Table A4).

**FIGURE 4:**
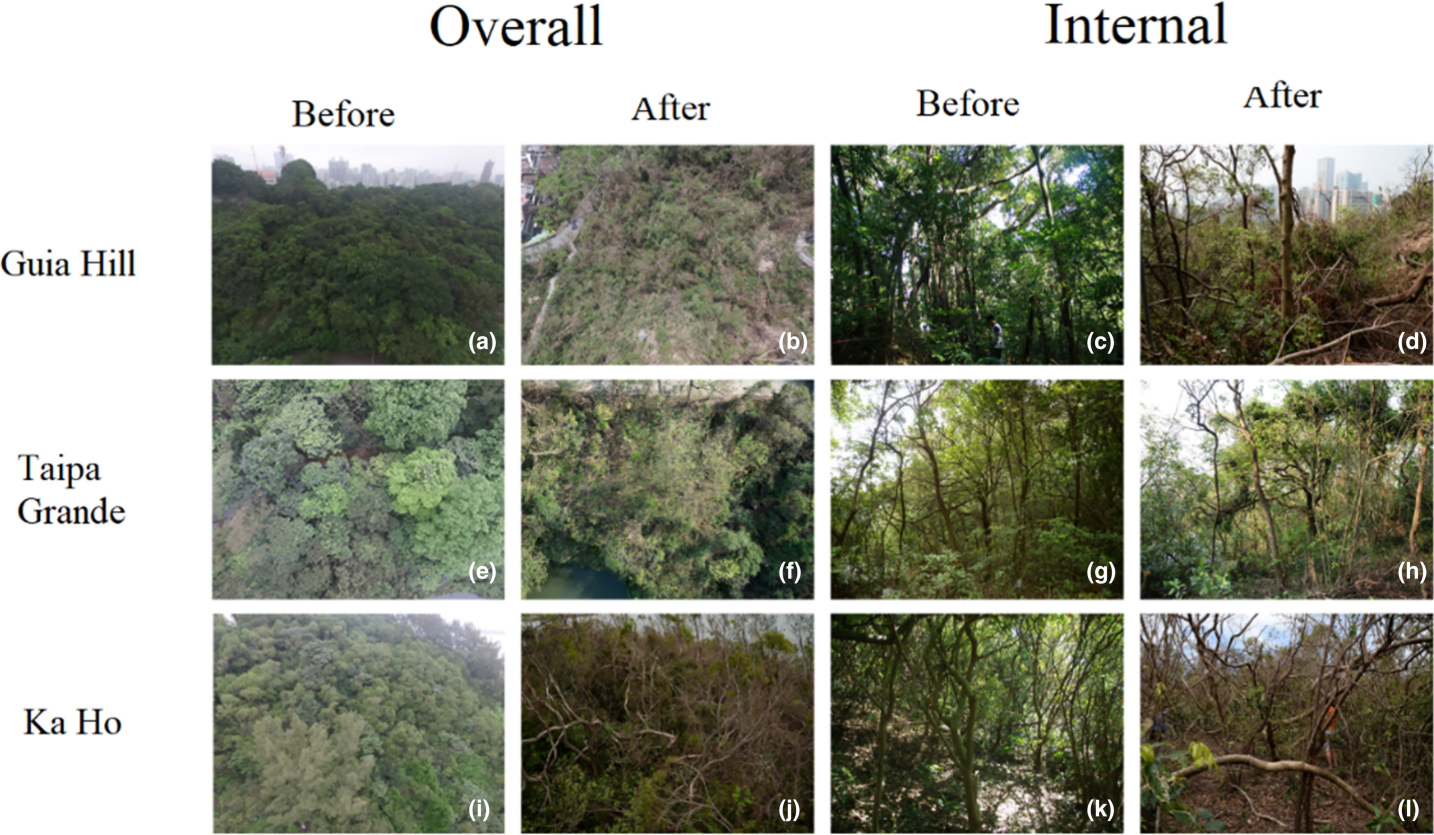
Representative pictures of overall (a,b, e,f, and i,j) and internal (c,d, g,h, and k,l) views of the plots before (Guia Hill: a, c; Taipa Grande: e, g; Ka Ho: i, k) and after (Guia Hill: b,d; Taipa Grande: f,h; Ka Ho: j,l) the typhoon Hato.

Our onsite field investigation revealed that the sub‐climax community of Guia Hill suffered the most serious disturbance from Hato (Figure 4a–d). The canopy was most affected by fallen trees, branches, and leaves covering the sample plot. Plants in the lower layers showed varying degrees of damage, but most leaves were left with withered margins. For the understory layer, the secondary injury is the main damage. At Ka Ho (Figure 4i–l), the trees were left with sparse branches and leaves, while most of the remaining attached leaves had withered margins. The managed forest of Taipa Grande had the least effects of the three sample plots (Figure 4e–h).

According to the damage index, Guia Hill was the most affected sample plot. 581 individuals, or 92.08% of all the plants, were affected (Table A5). The DG of the whole plot was 3.32, and the DI was 55.28%. 42.79% of stems in Guia Hill were severe affected (Degree 4–6), while stems showing Degree 3 damage were the most numerous, with 189 individuals (29.95%; Table A5). For Ka Ho, there were more individuals (263 plants, or 97.77%) influenced by the wind than in the other two plots. However, from the severity point of view, Ka Ho was less affected than Guia Hill, with fewer stems classified as Degree 4–6 (BR of 21.93%). More plants fell into lower degrees of damage (Degree 1–3, 204 plants, 75.84%) instead, and Degree 3 was the most abundant with 129 plants (47.96%) (Table A5).

In Taipa Grande, there were only 22 individuals found in Degrees 4–6. Thus, the BR was 14.77%. We also recorded 18 plants (12.08%) as having no damage (Degree 0) (Table A5).

### 3.2 The Hato effects on dominant species

Foster (1998) demonstrated that tree species varied in their ability to resist typhoon winds and survive. In this section, we chose the main tree species (no less than five individuals) of the community to analyze their effects (Table A6).

In Guia Hill, the average damage degree varied with species. Three species had DG > 4.0, which means the whole population was seriously affected by the typhoon, including *T. cochinchinensis* (5.7), *M. paniculatus* (4.4), and *C. burmannii* (4.1) (Table A6). Not only the DG, but the BRs were also high for these three species. Especially for *T. cochinchinensis*, all individuals were in degrees 5 and 6, with at least trunk damage after the typhoon. In the field investigation, we found that the degree 5 *T. cochinchinensis* was snapped at about 2/3 their height, but still with some side branches alive, while the degree 6 individuals were broken at the trunks and lost all the branches and leaves. However, the 1‐year‐later re‐investigation found that all of the *T. cochinchinensis* sprouted some new leaves, and especially degree 5 individuals recovered well. Field investigation also found *F. macrocarpa* and *L. monopetala* had new shoots sprout out. Not all species were seriously injured by Hato. There were three species whose DG ≤ 3.0, including *Aporosa dioica* (1.8), *Claoxylon indicum* (2.4), and *S. jambos* (3.0), meaning that in general these species were more wind resistant.

The species in Taipa Grande was less injured compared with the species in GuiaHill. The most seriously damaged species was *I. asprella*, but with only a DG of 3.2. Four species had DG under 3.0 (Table A6).

In Ka Ho, most species were also not seriously impacted after Hato. All the species analyzed had an average DG between 2.2 (*I. asprella*) and 3.7 (*A.a confusa*) (Table A6).

Several species had consistently low DG, even in different sample plots. Among the most common species, *S. lanceolata* incurred relatively light damage in all three plots, with 3.4 DG in Guia Hill, 2.8 in Ka Ho, and 1.8 in Taipa Grande (Table A6).

Besides, our field investigation also found some species were less affected than other species, such as *I. asprella*, *S. heptaphylla*, and *S. jambos*, with individuals ≥ 10. *S. superba*, *S. kwangtungense*, and *Aporusa dioica* with 5–10 individuals were also less effected species (Table A6).

Apart from the main tree species analyzed above, some canopy dominant tree species (large crown width but with a small number) and representative species in sub‐canopy and understory layers were not listed in Table A6, but are worth discussion. For instance, there was one *Ficus macrocarpa* with a large crown and many branches at the base of Guia Hill who suffered degree 6 damage during the typhoon. Although the main trunk and branches were blown down and the crown greatly decreased, some branches were still alive. As a fast‐growing species, it may recover quickly. In the sub‐canopy and understory layers, *P. asiatica* (total 180 individuals) and *D. chinensis* (40 individuals) in Guia Hill, and *P. asiatica* (20 individuals) in Ka Ho were the dominant species. These species were also less affected by the typhoon. Although there were individuals in all damage degrees, their DGs were low since their total number of trees was relatively large. The field investigation also found they mainly suffered secondary damage from the fallen trees or branches from the upper canopy.

### 3.3 Comparison of Hato effects across DBH classes

The distribution of plants with different DBH in the tree layers is an important indicator reflecting the stability of community structure (Fang et al., 2004). Degree 6 damage would have a greater impact on community composition and structure. Therefore, we would mainly report the situation of plants in degree 6 in different DBH classes in this section.

**FIGURE 5:**
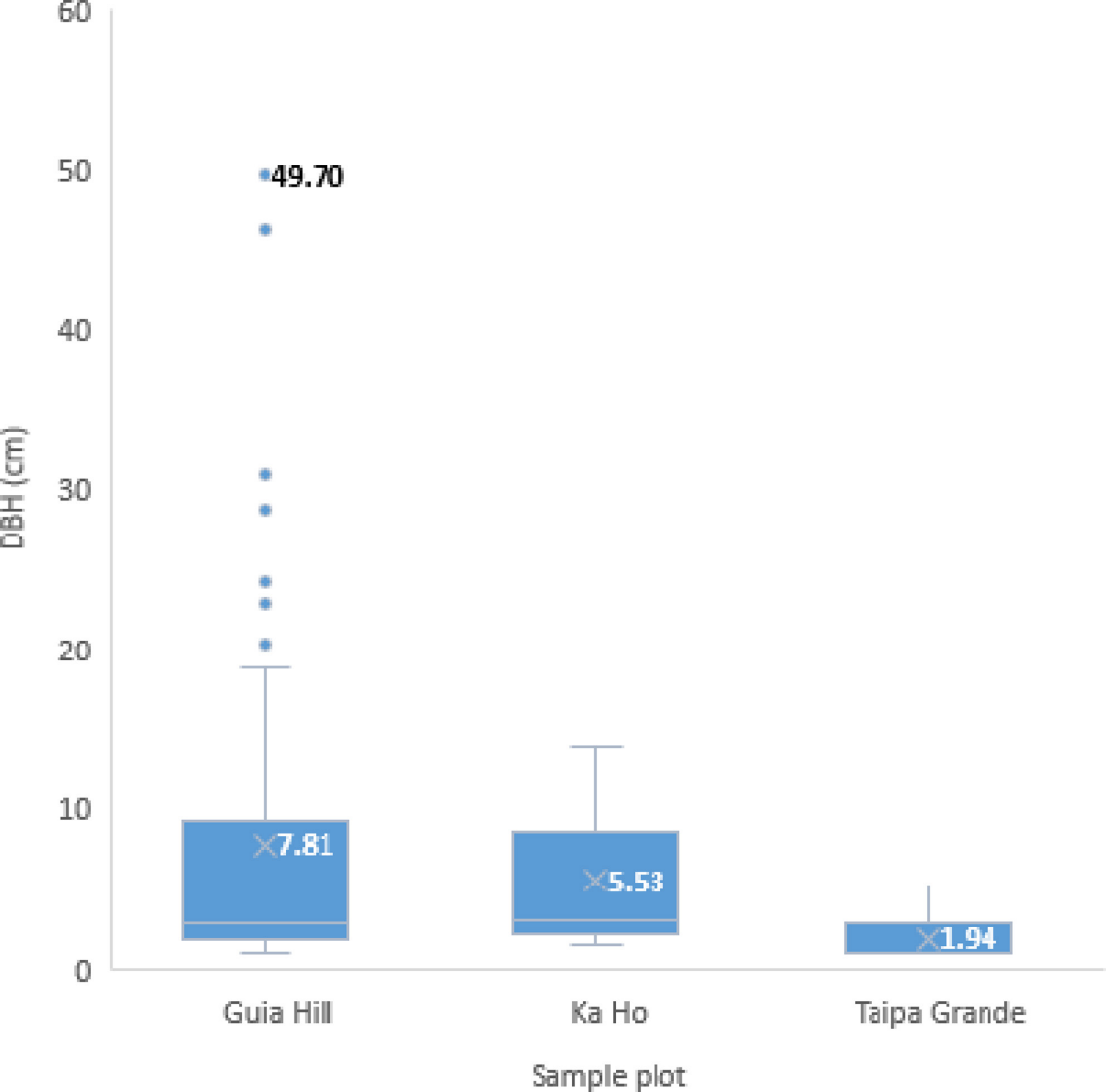
Boxplot of degree 6 tree DBH distribution (with average DBH indicated) for each sample plot.

Guia Hill had the most plants (87 individuals, or 13.79%) in degree 6, followed by Taipa Grande with 5.37% (8 plants) and Ka Ho with 4.46% (12 plants) (Table A5). We found that the largest average DBH of degree 6 damaged trees was in Guia Hill (7.81 cm) with a maximum DBH up to 49.7 cm, while the average DBH of degree 6 trees at Taipa Grande was the smallest (1.94 cm; Figure 5).

**FIGURE 6:**
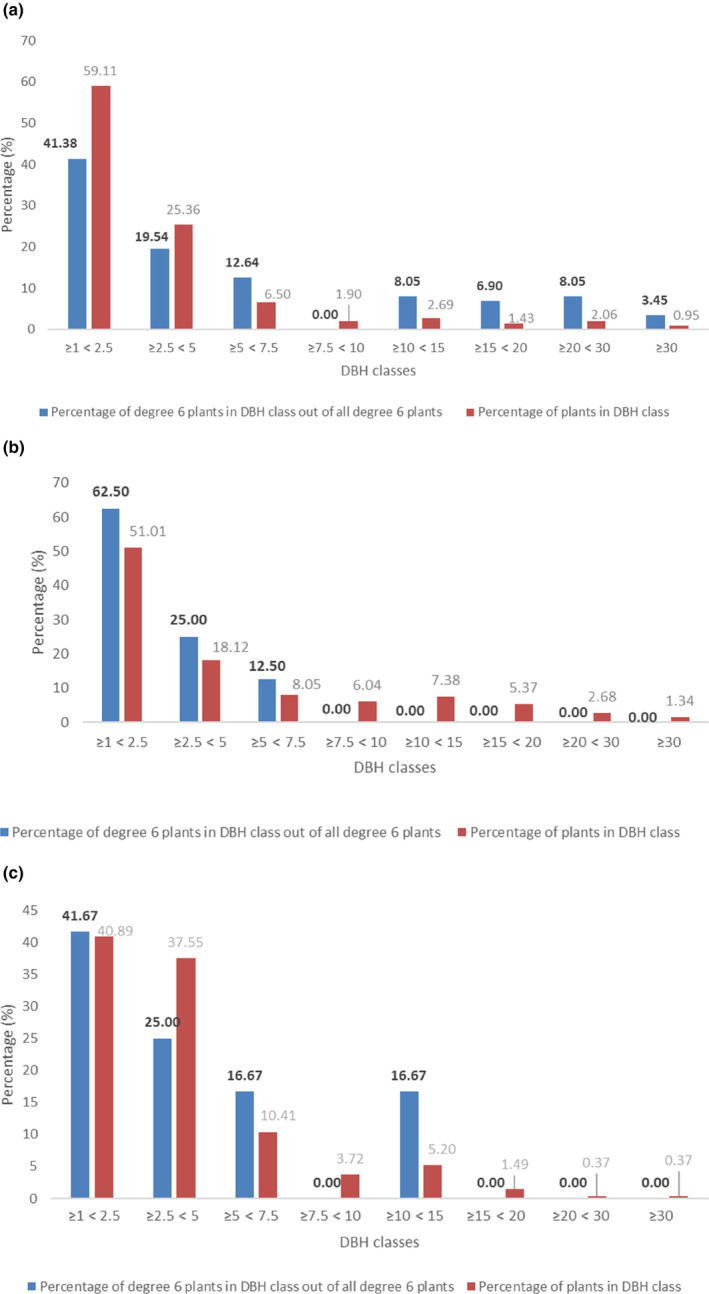
Bar chart of Degree 6 plants distribution among DBH classes for three plots (percentage of the Degree 6 individual number out of the total Degree 6 number in different DBH classes) and tree distribution among DBH classes for three plots (percentage of trees number out of the total trees in different DBH classes). (a: Guia Hill, b: Taipa Grande, c: Ka Ho).

To better understand which DBH classes have more degree 6 plants in three plots, degree 6 plant distribution (the proportion of degree 6 plants in the DBH class out of all degree 6 plants within the sample plot) and tree size distribution (the proportion of plants in the DBH class out of all plants) are shown in Figure 6. There were plants in all DBH classes in three sample plots. In general, around 41%–63% of the degree 6 plants were in the understory layer (1 cm ≤ DBH < 2.5 cm) in all three sample plots. Meanwhile, degree 6 individuals were also widely distributed in the sub‐canopy layer (2.5 cm ≤ DBH < 5 cm), accounting for 19.54% to 25% in three plots. However, the severely damaged plants with DBH ≥ 15 cm only appeared on Guia Hill.

**FIGURE 7:**
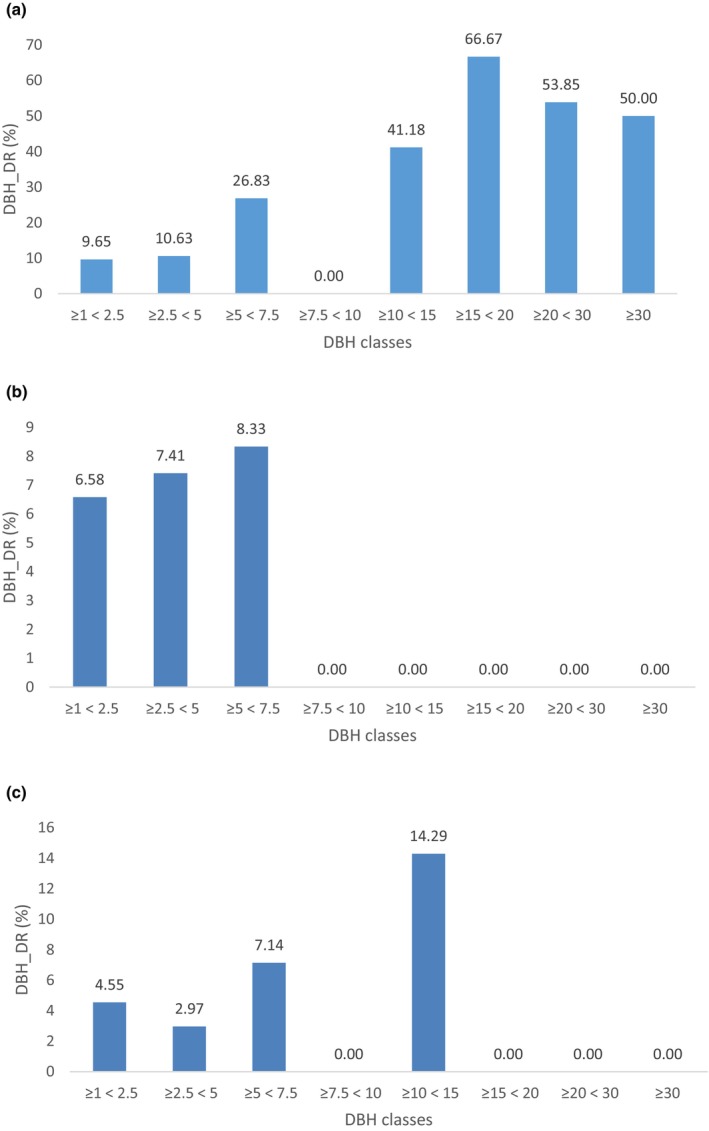
Bar chart DBH_DR of DBH classes in three sample plots. (a: Guia Hill, b: Taipa Grande, c: Ka Ho).

Guia Hill was the most affected plot since all DBH classes' DBH_DR ranged between 9.65% and 66.67%, which was higher than the max DBH_DR in Taipa Grande (8.33%) (Figure 7). In Guia Hill, three out of seven DBH classes had DBH_DRs of more than 50%. The maximum DBH_DR reached 66.67%, 4.6 times the maximum DBH_DR in Ka Ho (14.29%).

At Guia Hill, the plants with relatively large DBH were more likely to suffer severe damage. For example, the large DBH classes (DBH ≥ 30 cm, 20 cm ≤ DBH < 30 cm, and 15 cm ≤ DBH < 20 cm) had the highest three DBH_DR (50%, 53.85%, 66.67%) (Figure 7a). In Ka Ho, the most serious damage (DBH_DR of 14.29%) was in the class of 10 cm ≤ DBH < 15 cm, which was also the largest DBH class with Degree 6 trees in Ka Ho (Figure 7c). Taipa Grande acted the same with a max DBH_DR of 8.33% in the max class of 5 cm ≤ DBH < 7.5 cm (Figure 7b).

These data also indicated some different characteristics of degree 6 plants' DBH in the three sample plots. Trees with large diameters (10 cm ≤ DBH ~≥ 30 cm) were more likely to be damaged in Guia Hill, while the seriously damaged trees in Ka Ho had smaller DBH, and serious damages in Taipa Grande were limited to DBH < 7.5 cm. If classifying the DBH classes into tree layers, we could conclude that severe damage to the canopy layer was mainly encountered in Guia Hill and, to a lesser extent, at Ka Ho, while the sub‐canopy layer was most affected in Taipa Grande.

In conclusion, Guia Hill community structure was most impacted by Typhoon Hato since almost all DBH classes were damaged, and the large DBH trees were seriously damaged.

### 3.4 Comparison of dominant species before and after Hato among three sample plots

Our analysis of storm effects on the five most important species at each tree layer indicated some changes in community structure (Table A7).

On Guia Hill, the IV of *C. burmannii* decreased from 42.44 to 36.94 after Hato. Many large DBH individuals in *C. burmannii* died after the typhoon, reducing both the Breast Area (BA) and number of *C. burmannii,* leading to the lower IV. In contrast, the IV of *T. cochinchinensis* increased from 16.32 to 22.19 (Table A7). It was primarily because of the severe damage to other large DBH trees especially *C. burmannii*. The three next most important species changed little due to low mortality or minor structural effects.

Some of the top five species by IVs in the Ka Ho canopy also showed changes after the storm. *A. confusa* was the dominant species with a relatively large average BA and abundance in Ka Ho community, which results in its absolute dominance in the community. Although some *A. confusa* trees incurred severe damage from Hato, it was not enough to impact its IV rank. The IV of another dominant species in Ka Ho community, *L. rotundifolia* var. *oblongifolia*, decreased from 20.14 to 14.2. One of the characteristics of *L. rotundifolia* var. *oblongifolia* is that most trees are mutistemmed. In Ka Ho, lots of degree 4 and 5 *L. rotundifolia* var. *oblongifolia* stems were broken all degree 6 damaged individuals died since this species has weaker vitality than older trees. Therefore, the total RBA for this species decreased dramatically, leading to the IV decrease. However, the IV of *L. rotundifolia* var. *oblongifolia* was still ranked second. The relative decrease in IV for *L. rotundifolia* var. *oblongifolia* was accompanied by slight increases in IVs for *S. heptaphylla*, *M. paniculatus*, and *S. kwangtungense*, but their ranks remained the same.

In Taipa Grande, the IVs of the top five species in the canopy layer were similar before and after the typhoon.

Some species in the canopy layer had obvious changes in the following two special situations. One is that there were only a few individuals before the typhoon, and fewer individuals survived afterward, resulting in a significant decline of species' IVs and becoming associated species. One example would be *L. monopetala* on Guia Hill; there were two individuals in the canopy layer in total, and only one left after the typhoon. Another situation was the previously dominant species like *C. burmannii* and *L. rotundifolia* var. *oblongifolia* lost lots of large DBH individuals (broken or killed), with smaller BA individuals in the sub‐canopy or understory layer surviving. In general, the species' IVs and rank did not change much in these two layers in all three sample plots due to high species dominance. For example, in the lower two layers of Guia Hill, *C. burmannii* and *S. lanceolata* had a large number of individuals rank in the top 3. Therefore, after the storm effect, these two species IVs still rank in the top 3.

Typhoon Hato also led to the decline or disappearance of some species in these two lower layers. In Guia Hill, *Bridelia tomentosa* had one individual in the sub‐canopy layer with DBH of 5.5 cm that died in the typhoon., leaving one individual with 2.1 cm DBH alive in the understory layer.

## 4 DISCUSSION

Severe Typhoon Hato affected the local plant communities in Macau. Wind disturbance varied among three sample plots due to both biotic and abiotic factors. One of the biotic factors influencing differences in wind effects was forest age. Ni et al. (2021) found that the old growth forest was damaged more than twice as much as the secondary forest after being damaged by Typhoon Mangkhut. Mature forest communities have a high rate of stem breakage (Foster & Terborgh, 1998). Foster (1998) also found that wind damage to forests in central New England increases with forest age. Our study findings also confirmed the above research results. The oldest sub‐climax community, Guia Hill, suffered the most severe impact from Hato. Similarly, the Ka Ho mature shoreline shrub community also suffered considerable effects, while the managed, younger secondary forest around Taipa Grande was the least affected.

Community composition and structure also influenced storm effects. Strong winds selectively removed the oldest and tallest trees with large crowns in the forest (Li et al., 2018; Liu et al., 2012). Our results tie well with this view, especially for Guia Hill. The dominant species in the Guia Hill canopy layer were mainly taller and older trees, which were most affected. Also, dense forests tend to have a higher risk of damage and death when hit by strong winds (Mason & Quine, 1995; Stathers et al., 1994), which is the same case for the densest community, Guia Hill. Trees in the sub‐canopy and understory layer were more often injured by falling trees and broken branches, resulting in the deaths of small individuals in a restricted area around dominant tree (Xu et al., 2008). On Guia Hill, the canopy layer was seriously affected, meanwhile the sub‐canopy and understory layer were dense, so secondary damage was also common. By contrast, the dominant species in the canopy layer in Taipa Grande were shorter, with a smaller crown width. These species had a low lodging and breaking rate since they were less exposed to wind than tall individuals. Therefore, the secondary damage in the understory was also smaller than the effects in the Guia Hill understory. The coastal Ka Ho forest has many dwarfed and multibranched species, constructing a stable structure with dense branches intertwined with vines in between. This special structure was due to the naturally windy environment endemic to coastal islands (Peng, 2014). Therefore, the wind resistance of Ka Ho is relatively high, and trees were not easily damaged by the typhoon, and most of the trees only producing light litterfall.

Wind damage also varied by species, since wind resistance among different species has been shown to vary significantly (Bellingham et al., 1996; Gresham et al., 1991). Li et al. (2018) also indicated that different species have various sensitivities to wind damage. Our results lead to similar findings, where some species were less damaged by wind through all three sites. Among them, *P. asiatica* is distributed widely in the three sample plots, while *A. confusa* and *L. rotundifolia* var. *oblongifolia* were the dominant species of the Ka Ho. These less‐affected species are all local native trees. Their long‐term adaptation to the local environment makes them reliable as wind‐resistant species for Macau and surrounding coastal areas. Regular exposure to winds has likely exerted selective pressure on the traits of some tree species that are common in forests that frequently hit by typhoons. These environment‐selected wind‐resistant species could be recommended for the reconstruction of coastal communities similar to Macau that are frequently experience strong wind. Studies found that the severity and type of snow/wind damage for the community are related to individual tree attributes and stand‐level characteristics, and it is recommended to select suitable species for replanting in montane secondary forests (Li et al., 2018) and urban forests (Duryea et al., 2007; Hou et al., 2011).

One important finding of this study was that some small trees, such as *S. lanceolata*, *H. cochinchinense*, *I. asprella*, *M. paniculata*, *S. kwangtungense*, and *S. jambos*, mostly had broken branches and/or broken leaves instead of trunk damage, showing a potentially strong capability to be wind‐resistant tree species for restoration and enhancement of wind‐resistant of coastal urban forests in the future.

This study further found that although some species suffered the same in terms of the damage degree, the likelihood of survival and future recovery differed. Among the tall trees, degree 6 *C. burmannii* on Guia Hill were all leaning, broken at the base, or uprooted. The one‐year post‐storm census observed that those trees were dead. Some other species with the same height located at Guia Hill, such as *F. macrocarpa*, *L. monopetala*, and *T. cochinchinensis*, also incurred degree 6 damage, but new shoots had developed within 1 year, which indicated a higher possibility of recovery, similar to other findings (e.g. Van Bloem et al., 2007).

Everham and Brokaw (1996) concluded that the severity of wind damage to sample plots was affected not only by biotic factors but also abiotic factors. Many studies have reported that trees in exposed positions and on steep slopes (Tian et al., 2020) and at high altitudes (Martínez et al., 2013) are among the most susceptible to wind damage. Trees on windward surfaces or protruding ridges exposed to strong winds can incur substantial damage (Gao et al., 2019; Putz & Sharitz, 1991; Xi & Peet, 2008). Chen (2015) specifically analyzed Typhoon 1409 Wilmason and confirmed the wind damage was most severe at the top of the slope, followed by the middle of the slope, while the bottom of the slope suffered less. It was specified that the damage severity of rubber plantations at the windward slope, crosswind slope, and leeward slope decrease in sequence. Our study demonstrated that these abiotic factors also have an influence on the varied effects among the three plots. Guia Hill, which suffered the most, was situated near the top of a mountain; its terrain was slightly convex, and the slope was toward the south by southeast, parallel to the wind direction of this typhoon. These topographic factors likely had a combined effect on the vegetation of the plot, leading to more severe effects. Ka Ho was located along the seaside, close to the water. The terrain is slightly convex, sloped southward, facing the direction of the wind (windward slope) from the typhoon. The DR was also high there. Taipa Grande is located on a mountainside. It has valley‐like terrain that is slightly concave, and it is sloped northward to the northeast so as to block winds from the typhoon (leeward slope). The disturbance was lighter than Guia Hill. This topography factor influence found in this study could be compared to other typhoon‐affected case in the future.

## AUTHOR CONTRIBUTIONS

**Qifei Yi:** Conceptualization (lead); data curation (lead); formal analysis (lead); funding acquisition (lead); investigation (lead); methodology (lead); project administration (lead); resources (lead); software (lead); supervision (lead); validation (lead); visualization (lead); writing – original draft (lead); writing – review and editing (lead). **Wen Ye:** Investigation (equal); writing – review and editing (equal). **Faguo Wang:** Investigation (equal). **Fuwu Xing:** Conceptualization (supporting). **AJ Harris:** Writing – review and editing (supporting). **Lei Duan:** Writing – review and editing (supporting). **Hongfeng Chen:** Writing – review and editing (supporting).

## CONFLICT OF INTEREST STATEMENT

The authors declare no conflicts of interest, including financial and nonfinancial interests.

## Data Availability

All data generated or analyzed during this study are available on Figshare. Figshare DOI: 10.6084/m9.figshare.19311146.

## APPENDIX A

**TABLE A1:** General characteristics of sample plots.

Sample plot	Community type	Location	Age of community formation	Density (number per km^2^)	Height (m)	Aspect	Slope (degrees)
Guia Hill	Sub‐climax community	Macao Peninsula	≥100	7900	90	South to southeast	50
Taipa Grande	Artificial‐assistant‐restoration mesophytic community	Taipa	*ca*. 50	3800	129	North to northeast	40
Ka Ho	Shoreline shrub community	Coloane	50–60	9000	30	South to southeast	55

**TABLE A2:** The meteorological data of the typhoon Hato in the three sample plots (obtained from: http://www.smg.gov.mo/smg/introduction/c_aws_stations.htm).

Sample plot	Place	Wind direction	Hourly, Maximum wind speed (km/h)	Maximum gust (km/h)	Rainfall (mm)
Guia Hill	Macao Peninsula	South to southeast	113.8	197.3	30.8
Taipa Grande	Taipa	North	95.1	217.4	35.0
Ka Ho	Coloane	South	111.1	207.4	24.0

**TABLE A3:** Chi‐square test result for Damage Degree difference between sample plots

Chi‐Square tests			
	Value	df	Asymp. Sig. (2‐sided)
Pearson chi‐square	185.697^a^	12	0.000
Likelihood ratio	177.052	12	0.000
Linear‐by‐linear association	23.099	1	0.000
N of valid cases	1049		

^a^ 0 cells (0.0%) have expected count less than 5. The minimum expected count is 10.51.

**TABLE A4:** Pairwise *Z*‐tests result for Damage Degree difference between sample plots.

Damage Degree^a^ sampling plot crosstabulation					
		Sampling plot			
		Guia Hill	Taipa Grande	Ka Ho	Total
Damage Degree					
0.00	Count	50a	18a	6b	74
	% within Sampling Plot	7.9%	12.1%	2.2%	7.1%
1.00	Count	57a	60b	38a	155
	% within Sampling Plot	9.0%	40.3%	14.1%	14.8%
2.00	Count	65a	28b	37a, b	130
	% within Sampling Plot	10.3%	18.8%	13.8%	12.4%
3.00	Count	189a	21b	129c	339
	% within Sampling Plot	30.0%	14.1%	48.0%	32.3%
4.00	Count	98a	5b	29a	132
	% within Sampling Plot	15.5%	3.4%	10.8%	12.6%
5.00	Count	85a	9b	18b	112
	% within Sampling Plot	13.5%	6.0%	6.7%	10.7%
6.00	Count	87a	8b	12b	107
	% within Sampling Plot	13.8%	5.4%	4.5%	10.2%
Total	Count	631	149	269	1049
	% within Sampling Plot	100.0%	100.0%	100.0%	100.0%

*Note*: Each subscript letter denotes a subset of Sampling Plot categories whose column proportions do not differ significantly from each other at the .05 level.

^a^ For the same degree (each row), the comparisons of damage were made between sample plots. The different letter indicates in the specific the sample plots' effect was significantly differ than other plots in that degree.

**TABLE A5:** Statistics on damages caused by Typhoon Hato in sample plots (DG stands for the average damage degree, DR stands for the rate of wind damage, BR stands for the rate of severe damage caused by the typhoon, and DI stands for damage index).

Sample plot	Species	Number of plants	Number of plants/percentage of affected plants (%)							DG	DR (%)	BR (%)	DI (%)
			Degree 6	Degree 5	Degree 4	Degree 3	Degree 2	Degree 1	Degree 0				
Guia Hill	31	631	87/13.79	85/13.47	98/15.53	189/29.95	65/10.3	57/9.03	50/7.92	3.32	92.08	42.79	55.28
Taipa Grande	27	149	8/5.37	9/6.04	5/3.36	21/14.09	28/18.79	60/40.27	18/12.08	1.96	87.92	14.77	32.66
Ka Ho	27	269	12/4.46	18/6.69	29/10.78	129/47.96	37/13.75	38/14.13	6/2.23	2.89	97.77	21.93	48.14

**TABLE A6:** The damage of the canopy species whose total number was more or equal to five individuals.

	Species	DG	BR (%)	Degree 6	Degree 5	Degree 4	Degree 3	Degree 2	Degree 1	Degree 0
Guia Hill	*Triadica cochinchinensis*	5.7	100.0	4	2	0	0	0	0	0
	*Mallotus paniculatus*	4.4	72.7	6	11	7	8	1	0	0
	*Cinnamomum burmannii*	4.1	60.0	20	20	8	22	5	3	2
	*Microcos paniculata*	3.8	53.8	1	3	3	5	0	1	0
	*Syzygium levinei*	3.7	57.1	0	2	2	2	1	0	0
	*Sterculia lanceolata*	3.4	42.2	16	13	20	41	12	10	4
	*Schefflera heptaphylla*	3.1	25.0	2	0	1	6	1	1	1
	*Syzygium jambos*	3.0	37.8	1	6	7	9	7	5	2
	*Claoxylon indicum*	2.4	20.0	0	0	1	2	1	0	1
	*Aporosa dioica*	1.8	40.0	0	0	2	0	0	1	2
Taipa Grande	*Ilex asprella*	3.2	50.0	1	3	1	0	2	3	0
	*Litsea rotundifolia* var. *oblongifolia*	2.6	30.8	1	3	0	1	2	6	0
	*Schima superba*	2.4	12.5	0	0	1	2	4	1	0
	*Sterculia lanceolata*	1.8	9.8	2	0	2	4	9	22	2
	*Archidendron lucidum*	1.6	10.5	2	0	0	1	5	5	6
Ka Ho	*Acacia confusa*	3.7	48.3	2	5	7	13	2	0	0
	*Mallotus paniculatus*	3.3	21.7	2	0	3	17	1	0	0
	*Schefflera heptaphylla*	3.3	41.7	1	2	2	3	3	1	0
	*Litsea rotundifolia* var. *oblongifolia*	3.2	22.8	5	9	9	60	15	3	0
	*Eurya nitida*	3.1	30.0	0	1	2	5	1	1	0
	*Syzygium kwangtungense*	3.0	0.0	0	0	0	6	0	0	0
	*Sterculia lanceolata*	2.8	27.8	2	0	3	8	2	2	1
	*Ilex asprella*	2.2	0.0	0	0	0	3	0	2	0

**TABLE A7:** The top 5 species of relative importance values in three layers before and after Typhoon Hato in sample plots (IV‐After/IV‐Before stands for relative importance value after Typhoon Hato/before Typhoon Hato).

Tree layer	Guia Hill			Taipa Crande			Ka Ho		
	Species	IV‐After	IV‐Before	Species	IV‐After	IV‐Before	Species	IV‐After	IV‐Before
Canopy layer DBH ≥ 7.5 cm	*Cinnamomum burmannii*	36.94	42.44	*Sterculia lanceolata*	22.7	23.07	*Acacia confusa*	57.29	58.04
	*Triadica cochinchinensis*	22.19	16.32	*Schima superba*	19.29	19.64	*Litsea rotundifolia* var. *oblongifolia*	14.20	20.14
	*Sterculia lanceolata*	11.66	10.81	*Triadica cochinchinensis*	14.68	12.92	*Schefflera heptaphylla*	10.65	8.79
	*Mallotus paniculatus*	7.42	7.09	*Triadica sebiferum*	8.55	8.94	*Mallotus paniculatus*	9.29	6.97
	*Toxicodendron succedaneum*	4.52	3.76	*Mallotus paniculatus*	7.98	8.16	*Syzygium kwangtungense*	8.56	6.05
Sub‐canopy layer 2.5 cm ≤ DBH < 7.5 cm	*Sterculia lanceolata*	24.67	20.73	*Sterculia lanceolata*	24.61	24.15	*Litsea rotundifolia* var. *oblongifolia*	34.32	36.12
	*Psychotria asiatica*	18.02	18.40	*Litsea rotundifolia* var. *oblongifolia*	17.16	18.49	*Mallotus paniculatus*	17.63	14.85
	*Cinnamomum burmannii*	13.37	12.75	*Ilex asprella*	8.70	8.64	*Acacia confusa*	9.88	11.27
	*Syzygium jambos*	13.12	11.80	*Abarema lucida*	7.97	8.12	*Eurya nitida*	6.78	6.18
	*Mallotus paniculatus*	7.20	9.67	*Gymnema sylvestre*	6.67	6.81	*Sterculia lanceolata*	6.77	6.54
Understory layer 1.0 cm ≤ DBH < 2.5 cm	*Psychotria asiatica*	31.47	30.09	*Sterculia lanceolata*	23.67	25.84	*Litsea rotundifolia* var. *oblongifolia*	27.59	28.30
	*Sterculia lanceolata*	16.93	15.72	*Abarema lucida*	16.21	16.02	*Psychotria asiatica*	16.70	15.00
	*Cinnamomum burmannii*	9.86	10.20	*Psychotria asiatica*	13.41	12.70	*Sterculia lanceolata*	11.51	10.65
	*Desmos chinensis*	7.91	9.20	*Ilex asprella*	7.48	7.81	*Embelia laeta*	5.36	6.84
	*Elaeagnus loureirii*	5.62	6.78	*Litsea rotundifolia* var. *oblongifolia*	6.62	6.18	*Mussaenda pubescens*	4.73	4.41
